# Supplementary material for: Robust cell tracking in epithelial tissues through identification of maximum common subgraphs
Source: J R Soc Interface. 2016 Nov;13(124):20160725. doi: 10.1098/rsif.2016.0725 (PMC5134023; doi:10.1098/rsif.2016.0725)
Supplement: Mathematical description of the MCSTracker algorithm [file rsif20160725supp1.pdf]

Supplementary Material for the paper 'Robust cell tracking in epithelial tissues through identification of maximum common subgraphs' in Journal of the Royal Society Interface

Jochen Kursawe[1,*], Rémi Bardenet[2], Jeremiah J. Zartman[3], Ruth E. Baker[1], Alexander G. Fletcher[4,5,*]

[1] Mathematical Institute, University of Oxford, Andrew Wiles Building, Radcliffe Observatory Quarter, Woodstock Road, Oxford, OX2 6GG, UK

[2] CNRS & CRIStAL, Université de Lille, 59651 Villeneuve d'Ascq, France

[3] Department of Chemical and Biomolecular Engineering, University of Notre Dame, 205D McCourtney Hall of Molecular Science and Engineering, Notre Dame, IN 46556, USA

[4] School of Mathematics and Statistics, University of Sheffield, Hicks Building, Hounsfield Road, Sheffield, S3 7RH, UK

[5] Bateson Centre, University of Sheffield, Sheffield, S10 2TN, UK

* E-mail: kursawe@maths.ox.ac.uk, a.g.fletcher@sheffield.ac.uk

Data accessibility: The datasets and code supporting this article are publicly available at `https://github.com/kursawe/MCSTracker`.

How to cite: When using this supplementary material, the code, or the included data, please cite the main article in Journal of the Royal Society Interface. The main article is available available at `http://dx.doi.org/10.1098/rsif.2016.0725`.

A conceptual overview of our cell tracking algorithm is given in the main text. Here, we provide a detailed description of each step of the algorithm is provided in the section 'Mathematical formulation'. The input to the algorithm is a set of segmented images obtained from a live-imaging microscopy data set of the apical surface of an epithelial cell sheet. For each image, the segmentation is assumed to have correctly identified which cells are adjacent and the locations of junctions where three or more cells meet. This information is used to generate a polygonal approximation to the cell tessellation (figure S1A, figure 2A).

Our algorithm tracks cells between each pair of consecutive images in three steps (figure

2). First, we use a maximum common subgraph (MCS) approach [S1, S2] to generate an initial bijection between the two images that includes every cell whose connections to its neighbours do not change between images, e.g. due to cell rearrangements (manuscript figure 2B). Second, we remove from the bijection any cells that have less than three isolated connections to other cells in the MCS (figure 2B-C), as well as isolated clusters of fewer than ten cells, since these cells are likely to have been matched incorrectly. Third, we extend the MCS to track any remaining cells that were not included in the bijection and we identify cell division and 'removal' (delamination, extrusion or death) events (figure 2D) through characteristic changes to the local cell network under these events.

In the first of the three steps shown in figure 2, the MCS is constructed by iterative extension from an initial seed. The full MCS is then constructed by iteratively adding cells after inspecting MCSs of the cells' extended neighbourhoods.

Mathematical formulation

Preliminaries We begin by introducing the graph theoretic terminology and notation [S3] used to describe our algorithm. We consider each pair of successive segmented images as vertex-labelled graphs[1] $G = (V, E)$ and $G' = (V', E')$, respectively. Here and throughout, we use a prime symbol $'$ to refer to the latter of the consecutive images. Each vertex in G or G' corresponds to one cell in the respective segmentation, and two vertices share an edge in the graph if the corresponding cells are adjacent. Throughout, we assume the graphs G and G' to be simple, planar and connected; we emphasise that these graphs represent the dual of the polygonal cell packing (figure S1A). These assumptions are reasonable in the case of simple epithelial cell sheets.

The vertex labelling of G is defined by three functions, $p_G : V \to \mathbb{N}$, $x_G : V \to \mathbb{R}$, $y_G : V \to \mathbb{R}$, and $r_G : V \to T_G$. For a vertex $v \in V$, we refer to $p_G(v)$, $x_G(v)$, $y_G(v)$, $r_G(v)$ as the *polygon number*, *x coordinate*, *y coordinate*, and *neighbour order* of v, respectively. For a given vertex, the polygon number is the number of neighbours of the corresponding cell, and the x and y coordinates are defined by the centroid of that cell. T_G is defined as the space of ordered sets of arbitrary length on V, and the neighbour order $r_G(v)$ is the set of vertices that are adjacent

[1] A *graph* is an ordered pair $G = (V, E)$, where $V \subseteq \mathbb{N}$ and $E \subseteq \{A \subseteq V : |A| = 2\}$. The elements of V and E are called the *vertices* and *edges* of G, respectively. Given a graph $G = (V, E)$, a *vertex labelling* is a function of V to a set of labels. With this function, G is called a *vertex-labelled* graph.

to v, ordered by the clockwise appearance of their corresponding cells in the segmented image with an arbitrary starting point. An overlay of a polygonal tessellation with the corresponding graph structure is shown in figure S1A.

Let ϕ be an isomorphism[2] from $A \subseteq V$ to $B \subseteq V'$ such that for all $v \in A$, we have $p_G(v) = p_{G'}(\phi(v))$ and for all $x, y \in A$, we have $\{x, y\} \in E \Leftrightarrow \{\phi(x), \phi(y)\} \in E'$. We call ϕ a *cell mapping* from G to G' and define the *size* of ϕ to be $|\phi| = |A|$. We say the cell mapping ϕ *preserves rotational order* if for each vertex $v \in A$ the images of $r_G(v) \cap A$ have identical cyclic order to the set $r_{G'}(\phi(v)) \cap B$.

Let S denote the set of cell mappings from subgraphs of G to subgraphs of G'. Suppose that $\phi_{MCS} \in S$ has maximum size, i.e. $|\phi_{MCS}| \geq |\phi| \;\; \forall \phi \in S$, and let $V_{MCS} \subseteq V$ denote the domain of ϕ_{MCS}. We call the subgraph induced[3] by V_{MCS} a *MCS* of G and G' (this may not be unique). A non-trivial, i.e. non-empty, MCS exists if there are two vertices $v \in V$ and $v' \in V'$ that have the same polygon number, which is always true in our test cases. Our definition of a MCS differs slightly from previous definitions since it requires equivalence of the polygon number in addition to equivalence of edges [S1, S4]. Note that the polygon number and degree[4] of a vertex may not coincide for cells at the tissue boundary (figure S1A). A MCS is said to *preserve rotational order* if ϕ_{MCS} preserves rotational order.

Suppose that G and G' have k MCSs that preserve rotational order, with associated cell mappings $\phi_1, \ldots, \phi_k$. Let V_c denote the set of vertices in V that are mapped to the same vertex in V' by every cell mapping $\phi_1, \ldots, \phi_k$, and let ϕ_c denote the restriction of ϕ_1 (or, equivalently, any of the cell mappings) to V_c. We call V_c the *conserved MCS* of G and G'. In contrast to MCSs, conserved MCSs are unique. Examples of MCSs and conserved MCSs are illustrated in figure S1B.

Construction of the conserved MCS

In general, finding a MCS between two graphs is an NP-hard problem [S1]. Here we adapt an efficient MCS detection algorithm [S2] by exploiting graph planarity to reduce computational

[2] Graphs $G = (V, E)$ and $G' = (V', E')$ are *isomorphic* if there exists a bijection $\phi : V \to V'$ such that, for each $x, y \in V$, we have $\{x, y\} \in E \Leftrightarrow \{\phi(x), \phi(y)\} \in E'$. We say that ϕ is an *isomorphism.*

[3] A graph $G' = (V', E')$ is a *subgraph* of $G = (V, E)$ if $V' \subseteq V$ and $E' \subseteq E$. The subgraph G' of G is *induced* by the vertices $A \subseteq V$ if it contains all edges whose endpoints are both in A.

[4] The *degree* of a vertex v of a graph $G = (V, E)$ is the number of incident edges, $\deg_G(v) = |\{w \in V : \{v, w\} \in E\}|$.

complexity. Instead of exploring all possible combinations of vertex-to-vertex matches [S2] we construct the conserved MCS iteratively by finding the MCSs of small subgraphs of G and G'. To describe this construction we make use of the following definitions.

For a graph $G = (V, E)$, we define the *extended neighbourhood*[5] of a vertex $v \in V$ to be the set $\Gamma_G^{(2)}(v) = \{w \in V : d_G(v, w) \leq 2\}$, where d_G denotes graph distance[6]. The extended neighbourhood contains v, all neighbours of v, and all second nearest neighbours of v. An example of an extended neighbourhood is illustrated in manuscript figure 3A as the set of highlighted blue and grey cells.

Let $\rho : A \to B$ be a cell mapping, $v \in V \setminus A$ and $v' \in V' \setminus B$ be vertices in successive graphs, and S_{LM}^{ρ} be the set of cell mappings whose domains lie in $\Gamma_G(v)$, whose images lie in V', which map v to v', and which map v_a to $\rho(v_a)$ for all $v_a \in A \cap \Gamma_G(v)$. Suppose that $\phi_{LM}^{\rho} \in S_{LM}^{\rho}$ has maximum size, i.e. $|\phi_{LM}^{\rho}| \geq |\phi| \;\; \forall \phi \in S_{LM}^{\rho}$, and let V_{LM} denote the domain of ϕ_{LM}. We call the subgraph induced by V_{LM}^{ρ} a *local MCS* (LM) of v and v' under ρ.

Further, let S_{ELM}^{ρ} be the set of cell mappings whose domains lie in $\Gamma_G^{(2)}(v)$, whose images lie in V', which map v to v', and which map v_a to $\rho(v_a)$ for all $v_a \in A \cap \Gamma_G^{(2)}(v)$. Suppose that $\phi_{ELM}^{\rho} \in S_{ELM}^{\rho}$ has maximum size, i.e. $|\phi_{ELM}^{\rho}| \geq |\phi| \;\; \forall \phi \in S_{ELM}^{\rho}$, and let V_{ELM} denote the domain of ϕ_{ELM}. We call the subgraph induced by V_{ELM}^{ρ} a *extended local MCS* (ELM) of v and v' under ρ.

Further, let S_{RLM}^{ρ} denote the set of cell mappings whose domains lie in the extended neighbourhood of v excluding v, whose images lie in V', and which map v_a to $\rho(v_a)$ for all $v_a \in A \cap \Gamma_G^{(2)}(v)$. Suppose that $\phi_{RLM}^{\rho} \in S_{RLM}^{\rho}$ has maximum size and let V_{RLM}^{ρ} denote the domain of ϕ_{RLM}^{ρ}. We call the subgraph induced by V_{RLM}^{ρ} a *reduced local MCS* (RLM) of v under ρ.

Finally, we say that $v' \in V' \setminus B$ is *mappable* to $v \in V \setminus A$ under ρ if $p_G(v) = p_{G'}(v')$, $d_G(w, v) = 1 \Leftrightarrow d_{G'}(\rho(w), v') = 1$ for all $w \in A$, and if $(x_G(v) - x_{G'}(v'))^2 + (y_G(v) - y_{G'}(v'))^2 < d_{\max}^2$, where throughout this paper we choose the threshold $d_{\max}$ to be ten times the average cell diameter in the tissue (defined as the square root of the average area of the polygonal approximations of the cells in the segmented microscopy image). The threshold $d_{\max}$ is used in our MCS finding algorithm to restrict any possible vertex pairings to those that are in physical

[5]The *neighbourhood* $\Gamma_G(v)$ contains all $u \in V$ for which $(u, v) \in E$.

[6]The *distance* $d_G(v, w)$ between two vertices v, w of a graph G is the number of edges in a shortest path connecting them. If no such path exists, then the distance is set equal to ∞.

proximity. This restriction reduces the size of the search space.

Initial step To construct the conserved MCS, we first define a cell mapping ϕ_1 between single vertices of the consecutive graphs (manuscript figure 3A). Formally, we search through vertices in V and V' to find $v_1 \in V$, $v'_1 \in V'$ such that the order[7] of any extended local MCS of v_1 and v'_1 under the cell mapping[8] $\phi_0 : \emptyset \to \emptyset$ is equal to $|\Gamma^2_G(v_1)|$ and, for any vertex $v'_2 \in V' \setminus \{v'_1\}$ that is mappable to v_1 under ϕ_0, the order of any extended local MCS of v_1 and v'_2 is strictly less than $|\Gamma^2_G(v_1)|$. If no such v_1 can be found, we relax our condition and instead search for any $v_1 \in V$, $v'_1 \in V'$ such that the order of any local MCS of v_1 and v'_1 under the cell mapping $\phi_0 : \emptyset \to \emptyset$ is equal to $|\Gamma_G(v_1)|$ and, for any vertex $v'_2 \in V' \setminus \{v'_1\}$ that is mappable to v_1 under ϕ_0, the order of any local MCS of v_1 and v'_2 is strictly less than $|\Gamma_G(v_1)|$. We then define a first cell mapping $\phi_1 : V_1 \to V'_1$ with $V_1 = \{v_1\}$, $V'_1 = \{v'_1\}$ and a first *set of inspected vertices* $V^{\text{ins}}_1 = \emptyset$. Since we wish to use the MCS to aid our cell tracking, the equivalence of the (extended) neighbourhoods of v_1 and v'_1 gives us confidence that the corresponding cells are correctly tracked under ϕ_1. If we cannot find an initial cell mapping, then the algorithm halts; this means that the cell connectivity changes so quickly that the neighbourhood of every cell differs between consecutive images.

Iterative extension Our next step is to iteratively construct a cell mapping $\phi_{\text{cell}} : V_{\text{cell}} \to V'_{\text{cell}}$ for the conserved MCS between G and G', as follows.

For $n = 1, 2, \ldots$, given a cell mapping $\phi_n : V_n \to V'_n$ and a set of already inspected vertices $V^{\text{ins}}_n \subseteq V$, we determine the set of vertices $S_n \subseteq \Gamma_G(V_n) \setminus V^{\text{ins}}_n$ with at least one mappable vertex in $V' \setminus V'_n$ under ϕ_n. If there are no such vertices ($S_n = \emptyset$), then we simply define $\phi_{n+1} = \phi_n$, $V_{n+1} = V_n$, $V'_{n+1} = V'_n$, and set $V^{\text{ins}}_{n+1} = \emptyset$. Otherwise, if there are such vertices ($S_n \neq \emptyset$), then we find a vertex $v_{n+1} \in S_n$ with a smallest set of mappable vertices $M'_{n+1} \subseteq V' \setminus V'_n$ under ϕ_n. We then find all RLMs of v_{n+1} under ϕ_n and, for each vertex $v'_m \in M'_{n+1}$, we find all ELMs of v_{n+1} and v'_m under ϕ_n. Next, we find if there is a vertex $v'_{n+1} \in M'_{n+1}$ for which all ELMs of v_{n+1} and v'_{n+1} are larger than all ELMs of v_{n+1} and $v'_m \in M'_{n+1} \setminus \{v'_{n+1}\}$, and larger than all RLMs of v_{n+1}. Finally, we distinguish between the cases (i) where v'_{n+1} exists and the ELM preserves rotational order or (ii) where v'_{n+1} either does not exist or it exists and the

[7]The *order* of G is the number of its vertices, $|V|$.

[8]Here and throughout, $\emptyset$ denotes the empty set.

corresponding ELM does not preserve the rotational order. If such a vertex v'_{n+1} exists, then we define a new cell mapping $\phi_{n+1} : V_n \cup \{v_{n+1}\} \to V'_n \cup \{v'_{n+1}\}$ such that $\phi_{n+1}(v_{n+1}) = v'_{n+1}$ and $\phi_{n+1}(v) = \phi_n(v)\ \forall v \in V_n$, and define a new set of inspected vertices $V^{\text{ins}}_{n+1} = V^{\text{ins}}_n$. If there is no such vertex $v'_{n+1} \in \Gamma_G(V_n) \setminus V^{\text{ins}}_n$, then we construct an extended set of inspected vertices $V^{\text{ins}}_{n+1} = V^{\text{ins}}_n \cup \{v_{n+1}\}$, and set $\phi_{n+1} = \phi_n$, $V_{n+1} = V_n$, and $V'_{n+1} = V'_n$. We then increment n and return to the start of the iteration. Note that at each iteration the algorithm proceeds even if there are no non-trivial ELMs or RLMs for a given vertex v_{n+1}.

Whenever we encounter the set of adjacent cells with at least one mappable vertex $S_n = \emptyset$ for two consecutive values of n, the iteration continues with altering the definition of this set to the set of unmapped cells which have at least one mappable vertex, $S_n \subseteq V \setminus (V^{\text{ins}}_n \cap V_n)$, thus removing the restriction of the search to connected subgraphs. As soon as at least one cell mapping has been added to the MCS under this weakened condition, we set $V^{ins}_n = \emptyset$ and again continue the iteration among adjacent cells only, i.e. using $S_n \subseteq \Gamma_G(V_n) \setminus V^{\text{ins}}_n$ and again restrict the addition of new cells to cells adjacent to V_n.

The iteration halts whenever $S_n = \emptyset$ for three consecutive values of n. We then define $\phi_{\text{cell}} = \phi_n$, $V_{\text{cell}} = V_n$ and $V'_{\text{cell}} = V'_n$. Manuscript figure 3B-C illustrates the cells considered when searching for the RLMs and ELMs of a given vertex.

Post-processing

The cell mapping ϕ_{cell} is intended to correctly track as many cells as possible between consecutive images. Nevertheless, it is possible that some members of V_{cell} may be tracked incorrectly, while the cell mapping may have excluded some vertices in V that could have been tracked correctly. To eliminate tracking errors and track cells that are not included in the conserved MCS, we construct a *tracking mapping*, ψ_{track}, from $\tilde{V}_{\text{track}} \subseteq V$ to $\tilde{V}'_{\text{track}} \subseteq V'$. We call a mapping $\psi : \tilde{V} \subseteq V \to \tilde{V}' \subseteq V'$ a *tracking mapping* if it is an isomorphism from $\tilde{V}$ to $\tilde{V}'$. In contrast to a cell mapping, a tracking mapping need not preserve polygon numbers or edges between vertices of the subgraphs induced by $\tilde{V}$ and $\tilde{V}'$.

We begin by defining a first tracking mapping $\psi_1 = \phi_{\text{cell}}$ from $\tilde{V}_1 = V_{\text{cell}}$ to $\tilde{V}'_1 = V'_{\text{cell}}$. In the following, we describe how we iteratively refine the tracking mapping by first removing vertices from the domain that we suspect to correspond to incorrectly tracked cells (manuscript

figure 2B-C), and then we add vertices to the domain to track cells that are not members of the MCS (manuscript figure 2D).

Removing weakly connected cells Let ψ be a tracking mapping from $\tilde{V} \subseteq V$ to $\tilde{V}' \subseteq V'$. We define $v \in \tilde{V}$ to be *weakly connected* with respect to ψ if the set $\Gamma_G(v) \cap \tilde{V}$ contains either exactly one vertex; or exactly two vertices that are not adjacent. We remove any weakly connected vertices from the tracking mapping since the corresponding cells may have been tracked incorrectly by the MCS (manuscript figure 2). Once the weakly connected cells have been removed, we remove any cells from the tracking mapping that belong to connected components[9] which lie within the support of the tracking mapping and that contain less than ten vertices.

In practice, we first find the set of vertices $\tilde{S}_w \subseteq \tilde{V}_1$ that are weakly connected with respect to ψ_1. Next, we let $\tilde{V}_2 = \tilde{V}_1 \setminus \tilde{S}_w$, $\tilde{V}_2' = \tilde{V}_1' \setminus \{\psi_1(w) : w \in \tilde{S}_w\}$, and define a new tracking mapping $\psi_2 : \tilde{V}_2 \to \tilde{V}_2'$ to be the restriction of ψ_1 to $\tilde{V}_2$. Note that this step accounts for the possibility that $\tilde{S}_w = \emptyset$; in this case, we simply have $\psi_2 = \psi_1$.

Further, we identify the set S_i of all vertices that belong to any connected component $\tilde{C}$ within the graph induced by $\tilde{V}_2$ and for which $|\tilde{C}| < 10$. Next, we let $\tilde{V}_3 = \tilde{V}_2 \setminus \tilde{S}_i$, $\tilde{V}_3' = \tilde{V}_2' \setminus \{\psi_2(w) : w \in \tilde{S}_i\}$, and define a new tracking mapping $\psi_3 : \tilde{V}_3 \to \tilde{V}_3'$ to be the restriction of ψ_2 to $\tilde{V}_3$.

The removal of weakly connected cells and small isolated connected components prevents tracking errors in our algorithm. The network structure occasionally allows small connected components to match cells that do not physically correspond to each other. The same is true for weakly connected cells which tend to lie on the boundary of the conserved MCS.

Adding cells that were not tracked by the MCS We next add cells to the tracking mapping. This is necessary since any cells that have undergone neighbour exchanges between the consecutive images may have changed their polygon numbers, or their adjacency to each other. This means that their corresponding vertices cannot be members of the conserved MCS, and so regions of cell neighbour exchanges will leave gaps of untracked cells in the MCS (manuscript figure 2B-C).

In the following, we iteratively extend the domain of the tracking mapping to include vertices

[9]A *connected component* $C \in V$ in $G = (V, E)$ is a set of vertices $C \in V$ such that all pairs of vertices $v_1, v_2 \in C$ are connected by paths in V and that has no connections to vertices outside C.

that have neighbours within the domain of the tracking mapping. Possible images of a given vertex can be identified by the aid of the images of the neighbours of the vertex. In this way, we track as many remaining cells as possible based on their neighbour relationships to cells that have been tracked by the conserved MCS. The more mapped neighbours that are preserved between a newly added vertex and its image, the higher our confidence that the corresponding cells are correctly tracked.

Formally, we start with a tracking mapping $\psi_n : \tilde{V}_n \to \tilde{V}'_n$ (initially with $n = 3$). For each vertex in $\tilde{V} \setminus \tilde{V}_n$ we define the *conserved connectivity* $c_n(v)$ as the maximal number of unique neighbours of v in $\tilde{V}_n$ whose images under ψ_n have exactly one shared neighbour v'. Since $c_n(v)$ is the maximal number of *unique* shared neighbours, we set $c_n(v) = 0$ if there are multiple sets of neighbours of $S_i(v)$ that have a exactly one shared neighbour $S_i(v')$ and whose size is maximal.

In practice, we determine $c_n(v)$ as follows. For a given vertex v in $\tilde{V} \setminus \tilde{V}_n$, let $T_n(v) = \{\psi_n(w) : w \in \Gamma_G(v) \cap \tilde{V}_n\}$ denote the set of images of all adjacent vertices of v in the domain of the current tracking mapping. If $|T_n(v)| < 2$ we let $c_n(v) = 0$. Otherwise, we construct the set of vertices in $V' \setminus \tilde{V}'_n$ that elements of $T_n(v)$ share as neighbours,

$$W_n^{(0)}(v) = \bigcup_{v' \in T_n(v)} \Gamma_{G'}(v') \setminus \tilde{V}'_n. \tag{1}$$

If $W_n^{(0)}(v)$ is empty and $|T_n(v)| > 2$, we consider reduced sets of images of the form $T_n(v) \setminus \{w'\}$, where one element w' is removed from $T_n(v)$, and we define the set of all shared neighbours of each reduced image set that are not in the image of ψ_n:

$$W_n^{(1)}(v) = \bigcup_{w' \in T_n(v)} \left(\bigcap_{v' \in T_n(v) \setminus \{w'\}} \Gamma_{G'}(v') \setminus \tilde{V}'_n \right). \tag{2}$$

By construction, the set $W_n^{(1)}(v)$ contains those vertices in $V' \setminus \tilde{V}'_n$ that are shared neighbours of images of neighbours of v, each excluding one such neighbour. If $W_n^{(1)}(v)$ is empty, we analogously define $W_n^{(2)}(v)$ as the set of vertices in $V' \setminus \tilde{V}'_n$ that are shared neighbours of images of neighbours of v, each excluding two such neighbours. If $W_n^{(2)}(v)$ is empty, we continue analogously to define $W_n^{(k)}(v)$ as long as $0 < k < |T_n(v)| - 2$. If (i) $W_n^{(k)}(v), 0 < k < |T_n(v)| - 2$ contains exactly one vertex v', then we consider v' to be a possible match of v and set $c_n(v) =$

$|T_n(v)| - k$ as the number of neighbours of v' that are images of neighbours of v under ψ_n. If no unique v' can be found, we set $c_n(v) = 0$.

If the maximal conserved connectivity $c_n(v)$ among all vertices $v \in \tilde{V} \setminus \tilde{V}_n$ is greater than 2, the algorithm attempts to find a vertex v and its unique possible match v' such that $c_n(v)$ is maximal and such that the number of neighbours of v' in $\tilde{V}'_n$ that are not images of neighbours of v is less than $c_n(v) - 2$. If no such vertex vertex can be found, the algorithm attempts to find a vertex and its possible image such that this number of gained gained mapped neighbours is less than $c_n(v) - 1$. We then let let $\tilde{V}_{n+1} = \tilde{V}_n \cup \{v\}$, $\tilde{V}'_n = \tilde{V}'_n \cup \{v'\}$ and define a new tracking mapping $\psi_{n+1} : \tilde{V}_{n+1} \to \tilde{V}'_{n+1}$ to be the extension of ψ_n for which $\psi_{n+1}(v) = v'$. The algorithm then increments n and attempts to find a next match v and v' with maximal conserved connectivity.

As soon as the maximal conserved connectivity $c_n(v)$ among all vertices $v \in \tilde{V} \setminus \tilde{V}_n$ is less than two or if no new pair v and v' can be identified, the algorithm halts.

Resolving division events If a cell divides between consecutive frames, then the tracking mapping ψ_n we have constructed thus far may incorrectly map the mother cell with one of its daughter cells (manuscript figure 4). To address this issue, we construct a tracking mapping ψ_{track} in which incorrectly tracked mother cells are removed. To resolve division events, we first identify *boundary vertices* to be those vertices $v \in V$ whose polygon number and degree differ. This corresponds to cells that are at the physical boundary of the sheet, where polygon number and network degree do not coincide (figure S1A). We then identify all connected sets of vertices $M' \subseteq V' \setminus \tilde{V}'_n$ that satisfy $\Gamma'_G(M') \subseteq \tilde{V}'_n$ and that contain no boundary vertices of V'. Each such set M' corresponds to one division event, and in the following we treat each M' individually.

For each M', we define $S_{M,1} = \psi_n^{-1}(\Gamma'_G(M'))$ to be the set of inverse images of the mapped neighbours of M' under ψ_n. Next, we identify the set $S_{\text{border}} \subseteq S_{M,1}$ of potential bordering cells of the division, i.e. cells that are adjacent to the division, by finding those vertices $v \in S_{M,1}$ that gain an edge under the tracking mapping ψ_n:

$$S_{\text{border}} = \{v \in S_{M,1} : p_{G'}(\psi_n(v)) = p_G(v) + 1\}. \tag{3}$$

We also identify the set S_{mother} of potential mother cells by finding any shared neighbours of

potential bordering cells:

$$S_{\text{mother}} = \bigcap_{v \in S_{\text{border}}} \Gamma_G(v). \tag{4}$$

Based on the sets S_{border} and S_{mother} we decide which cells are the mother and daughter cells of the division event, distinguishing between the following cases:

(i) If S_{mother} contains exactly one vertex, then this is identified as the mother cell of the division, and M' must contain exactly two vertices, which are identified as the daughter cells. In this case, neither the mother nor daughter cells are three- or four-sided.

(ii) If $S_{\text{mother}} = \emptyset$, then one of the daughter cells must be three-sided (manuscript figure 4C). In this case, a geometry-inferred selection of mother and daughter cells is required. To this end, we define a set of potential daughter cells,

$$S'_{\text{daughter}} = \psi_n(S_{\text{border}}) \bigcup \left(\bigcap_{v' \in \psi_n(S_{\text{border}})} \Gamma_{G'}(v') \right), \tag{5}$$

that contains the images of the potential bordering cells and all shared neighbours of these images in V'. Next, we find a definite daughter cell as an element $v' \in S_{\text{daughter}}$ that is three-sided ($p_{G'}(v) = 3$). The geometry-inferred selection of the second daughter cell proceeds as follows. For each $w' \in S'_{\text{daughter}} \setminus \{v'\}$, we construct the *geometrically merged cell* of v' and w' by removing the edge between the polygons that corresponds to v' and w' in the segmentation of the microscopy video frame from which the graph G' was generated, as well as the cell junctions where three or more cells meet at the end of this edge. We then calculate the distance of the centroid of the geometrically merged cell to the centroid of the cell associated with vertex $\psi_n^{-1}(w')$. The vertex w' for which this distance is minimal is identified as the second daughter cell, and the mother cell is identified as its inverse image under ψ_n.

(iii) If S_{mother} contains more than one vertex, then we define a set of potential daughter cells as any shared neighbours of images of the potential bordering cells

$$S'_{\text{daughter}} = \bigcap_{v' \in \psi_n(S_{\text{border}})} \Gamma_{G'}(v'). \tag{6}$$

If S'_{daughter} contains exactly four vertices, then the mother cell and both daughter cells

are four-sided, and the mother cell can be identified as the single vertex in the set $S_{M,2}$, which we define as the set of cells which are shared neighbours of all cells in $S_{M,1}$ (the inverse images of neighbours of the division), and which are not in the domain of ψ_n, i.e.

$$S_{M,2} = \bigcap_{v \in S_{M,1}} \Gamma_G(v) \setminus \tilde{V}_n. \tag{7}$$

The daughter cells correspond to the only two vertices in M'.

If S'_{daughter} contains exactly three vertices, then one of the daughter cells is four-sided, and we identify this cell as the definite daughter cell of the division v', i.e. we identify $v' \in S'_{\text{daughter}} : p_{G'}(v') = 4$. In this case, geometry-inferred selection of the second daughter cell is required, and we achieve this in a similar way to that described for three-sided daughter cells above. For each cell $w' \in S'_{\text{daughter}} \setminus \{v'\}$, we construct the merged cell of v' and w', and calculate the distance of its centroid to the centroid of $\psi_n^{-1}(w')$. The cell $w' \in S'_{\text{daughter}} \setminus \{v'\}$ for which this distance is smallest is identified as the second daughter cell. Since in this case S_{mother} contains more than one cell, S'_{daughter} must contain at least three cells[10].

Once each set M' has been inspected and the associated division event has been resolved by identifying the mother and daughter cells, we construct a tracking mapping in which any incorrectly tracked mother cells are removed. To this end, we define the set of all mother cells for which geometry-inferred selection has been used as S_{geo}, and we construct a final tracking mapping $\psi_{\text{track}} : \tilde{V}_n \setminus S_{\text{geo}} \to \tilde{V}'_n \setminus \psi_n(S_{\text{geo}})$ such that $\psi_{\text{track}}(v) = \psi_n(v)\ \forall v \in \tilde{V}_n \setminus \psi_n(S_{\text{geo}})$.

In general, the division resolution step may incorrectly track cells in cases where there is a cell neighbour exchange next to the division, or if there are two adjacent divisions between frames. For example, if each of the bordering cells, i.e. the cells adjacent to the division, were to undergo a neighbour exchange in which they lose an edge between images, then our algorithm would fail to correctly resolve the division event.

Resolving remaining events At this stage, the tracking algorithm for the two consecutive time frames is completed, and it is straightforward to identify cell neighbour exchanges by

[10]If S'_{daughter} contains more than four cells, then our algorithm fails; however, this was never encountered in our test cases.

finding any cells that have changed their polygon number from one frame to the next. Cell removal events correspond to any vertices $v \in V$ that are not in the domain of ψ_{track}, for which $\Gamma_G(v) \subseteq V_{\text{track}}$, and that do not correspond to mother cells of a division event.

Computational implementation

We use Krissinel's MCS finding algorithm [S2] to find all RLMs and ELMs in the above steps. This algorithm will always halt eventually. In particular, since the domains on which the RLMs and ELMs are calculated only contain extended neighbourhoods of individual cells, the MCS finding does not pose computational barriers. We adapt the procedure for MCS finding proposed in [S2] in two ways: (i) whenever a next vertex is considered for mapping, we pick a vertex that is adjacent to already mapped cells, hence the adapted algorithm only finds connected subgraphs; (ii) since the RLMs and ELMs are small, we do not implement subgraph-size dependent conditions to interrupt the search early.

When finding the initial mapping, for any two possible matches ELMs are first calculated by considering nearest neighbours only rather than extended neighbourhoods. Once the neighbourhoods[11] of two matching vertices are found to be isomorphic, the extended neighbourhood is considered. This step reduces the time that is needed to find the initial match. During the post-processing, the conserved connectivity is not calculated for each vertex. Instead, vertices with maximal number of neighbours in the support of ψ_n are observed first and once the maximal conserved connectivity and the set of corresponding vertices has been identified no further conserved connectivities are calculated. This reduces the computational cost of the post processing algorithm.

In the computational implementation of the tracking algorithm we use a further vertex-label $c_G : V \to \mathbb{N}$, which we call the *cell identifier*. In practice, integer identifiers for a given vertex v arise naturally in the segmentation step. Cell identifiers allow us to easily identify vertices and relate them to a cell in a given image independent of how they are stored in the graph structure.

[11] The set of adjacent vertices, $\Gamma_G(v) = \{w \in V : \{v, w\} \in E\}$ is called the *neighbourhood* of v, so the degree of v is $|\Gamma_G(v)|$. We define the neighbourhood of a subset $V' \subseteq V$ to be $\Gamma_G(V') = \{w \in V \backslash V' : \exists\, v \in V' \text{ with } d(w, v) = 1\}$.

References

[S1] Ullmann, J. R. An algorithm for subgraph isomorphism. JACM, 23(1):31–42, 1976. 10.1145/321921.321925.

[S2] Krissinel, E. B. and Henrick, K. Common subgraph isomorphism detection by backtracking search. Software Pract. Exper., 34(6):591–607, 2004. 10.1002/spe.588.

[S3] Wilson, R. An Introduction to Graph Theory. Prentice Hall, 5th edition, 2010.

[S4] Raymond, J. W. and Willett, P. Maximum common subgraph isomorphism algorithms for the matching of chemical structures. J. Comput.-Aided Mol. Des., 16(7):521–533, 2002. 10.1023/A:1021271615909.

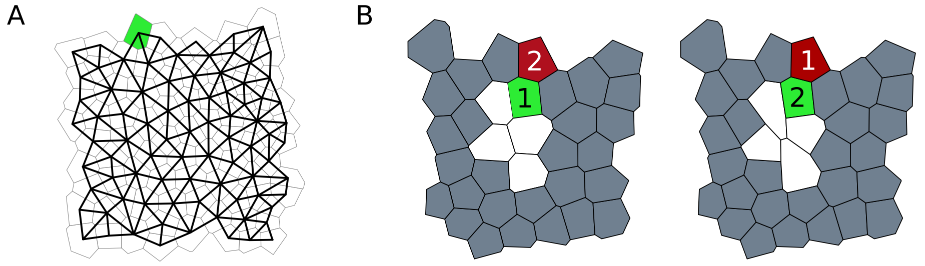

Figure S1: Construction of the MCS. (A) Overlay of a polygonal tessellation (grey) and the corresponding cell network (black). Each cell corresponds to one vertex in the network, and two vertices share an edge if the corresponding cells are adjacent. The network of cells is used by the algorithm to determine the MCS between tessellations corresponding to consecutive time frames in a microscopy video. Note that the network degree of a cell and its polygon number differ at the boundary of the tissue. For example, the highlighted cell has polygon number five and network degree three. (B) The dark grey cells are members of the conserved MCS between the two *in silico* tissues. In this example, two distinct MCSs are possible. Both MCSs include all highlighted grey, green, and red cells. The two MCSs differ in the way the numbered cells are mapped. The first MCS includes the cell pairings as indicated by the green (light) and red (dark) cells. The second MCS includes the pairings as indicated by the numbers 1 and 2. White cells are not members of the two MCSs.
